# Investing to gain others’ trust: Cognitive abstraction increases prosocial behavior and trust received from others

**DOI:** 10.1371/journal.pone.0284500

**Published:** 2023-04-14

**Authors:** Gijs van Houwelingen, Marius van Dijke

**Affiliations:** 1 Faculty of Economics and Business, University of Amsterdam, Amsterdam, the Netherlands; 2 Rotterdam School of Management, Erasmus University Rotterdam, Rotterdam, the Netherlands & Nottingham Business School, Nottingham Trent University, Nottingham, United Kingdom; UMIT TIROL - Private University for Health Sciences and Health Technology, AUSTRIA

## Abstract

Being trusted has many positive implications for one’s wellbeing (e.g., a better career, more satisfying interpersonal relationships). Scholars have suggested that people actively attempt to earn trust. However, it is not clear what makes people invest in actions that may earn them trust. We propose that cognitive abstraction (more than concreteness) facilitates seeing the long-term benefits of performing behaviors (i.e., prosocial behaviors) for gaining trust. We conducted a survey among employees and their supervisors and two yoked experiments—total *N* = 1098 or 549 pairs. In support of our claim, we find that cognitive abstraction leads to more prosocial behavior, which subsequently increases trust received. Furthermore, the effect of abstraction on the performance of prosocial behavior is limited to situations where such behavior can be observed by others (and thus be a basis for gaining observers’ trust). Our research shows when and why people decide to act in ways that may gain them trust and clarifies how cognitive abstraction influences the display of prosocial behavior and the subsequent trust received from fellow organization members.

## Introduction

Being trusted (vs. being monitored closely or being kept at a distance) results in receiving greater resources and opportunities. For instance, in work contexts, highly trusted organization members perform better, have more succesful careers, and experience higher wellbeing than non-trusted members [1–6]. Some scholars have suggested that because trust is an important form of social capital [7], individuals may actively attempt to build or preserve trust [8–10].

**Fig 1:**
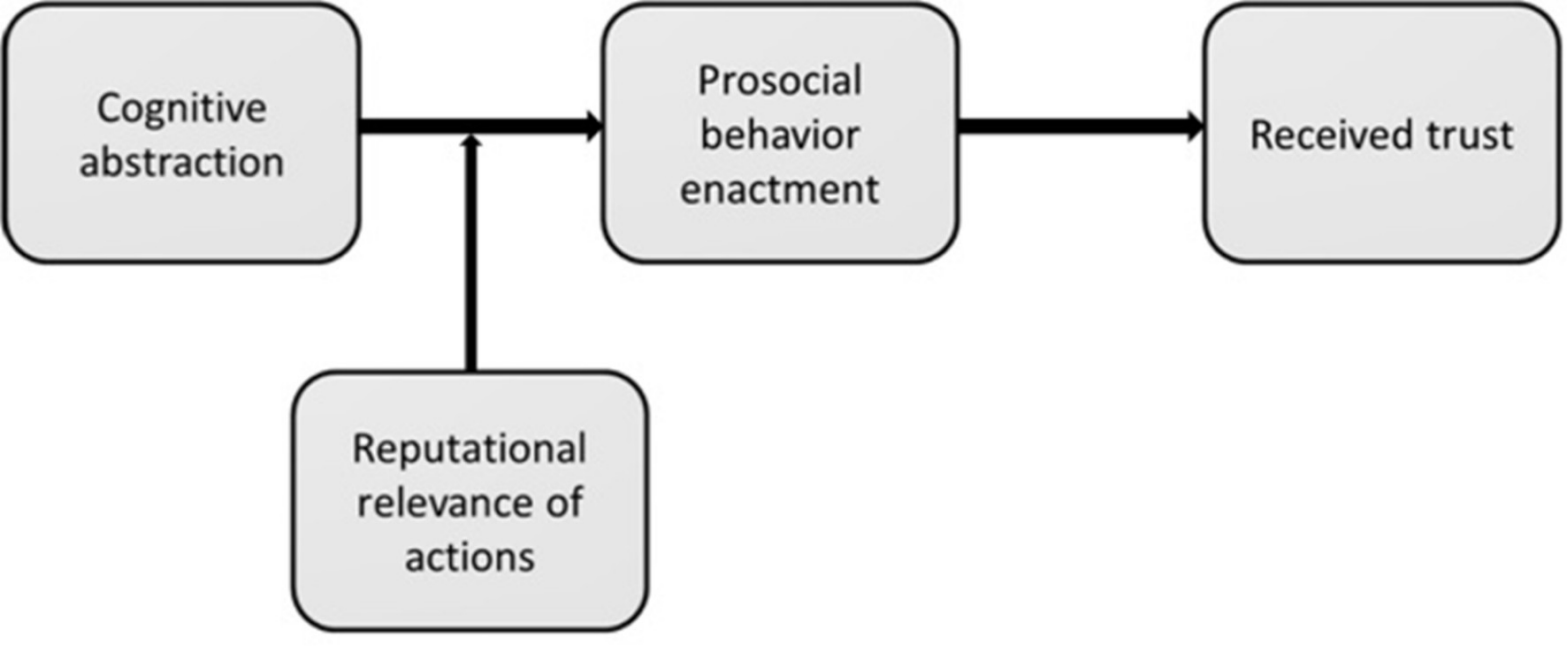
Theoretical model.

It is unclear when and why people invest in actions that may gain them trust. Our present research aims to answer this question. Investing in gaining trust implies orienting oneself towards an abstract goal (i.e., gaining and maintaining a trustworthy reputation) and that the benefits that result from being trusted are mostly immaterial and accumulate over time [11, 12]. Therefore, people need to be able to connect their behavior to obtaining such abstract benefits. Given this, we propose that *cognitive abstraction* (vs. concreteness) facilitates gaining trust. This is because cognitive abstraction (vs. concreteness) facilitates traversing mental distances [13] and, in this manner, allows people to more clearly see how their behavior is connected with their long-term goals [14]. More specifically, we predict that cognitive abstraction facilitates the display of prosocial behavior, which is an established antecedent of trust from the targets of proscial behavior and from observers alike [15–17]. Furthermore, because we expect cognitive abstraction to encourage prosociality mostly as an investment in trust, we also hypothesize that the effect of cognitive abstraction on prosocial behavior and therefore, on trust, will be diminished when situations do not (vs. do) carry reputational relevance. Fig 1 represents our model. We test our predictions in a survey among leader–employee pairs in work organizations and in two controlled experiments.

We identify cognitive abstraction as a factor that makes people invest in actions that may gain them trust. In doing so, we contribute to the literature on active trust, that is, the study of people’s actions that are intended to improve, build, or preserve trust [7, 8, 10, 18]. The literature on active trust has mostly focused on motives that inspire trustworthy behaviors [19]. We show that people may not always be able to spot opportunities to gain trust (i.e., when they construe matters at concrete levels).

Cognitive abstraction has increasingly become a variable of interest to social scientists. We address two puzzles in this literature. First, up to now it is unclear whether and how abstraction relates to being trusted [20]. Second, it has been suggested that abstraction may increase prosocial behavior [21]; however, there is only indirect empirical support for this claim. Prior work shows that cognitive abstraction (vs. concreteness) makes people apply justice principles to distant others [22], increases the anticipated reward of performing prosocial behavior [16], and strengthens prosocial attitudes [23]. However, given the well-established gap between attitudes and behaviors [24, 25], it is important to show that cognitive abstraction increases actual prosocial behavior and subsequently, the trust received. Our research is the first to show when and why cognitive abstraction promotes prosocial behavior.

### Theory development

#### Active trust and reputation

Trust is commonly defined as “a psychological state comprising the intention to accept vulnerability based upon positive expectations of the intentions or behavior of another” [26] (see also [27]). Accepting vulnerability is crucial in settings in which people depend on others to be able to function effectively [28]. For instance, organization members are typically motivated to pay close attention to the characteristics and behaviors of (potential) trustees to decide whether accepting vulnerability is safe [29].

Trust is an important form of social capital [30]: being trusted comes with many benefits [1]. Accordingly, trustees may actively attempt to build or preserve trust [8–10]. They can do so by displaying behaviors that signal trustworthiness [31, 32], particularly prosocial behaviors [16]. Prosocial behaviors usually have few immediate benefits for the actor (apart from received recognition or a warm glow [33, 34]) but are valuable for others (e.g., helping behavior [35]) [35]. Performing prosocial behaviors is therefore an ideal way to signal trustworthiness to the targets and onlookers of one’s prosocial behavior because such behavior signals unselfishness and thus, a lower likelihood of taking advantage of the vulnerabilities of others [36]. Consequently, through prosocial behaviors, one may earn trust of both targets and onlookers [6, 15, 37].

In sum, most benefits that result from performing prosocial behavior materialize only in the long run from having established a trustworthy reputation [7]. Therefore, a person who recognizes how their behavior is connected to obtaining such an abstract benefit (vs. not) should make better use of opportunities to establish for themselves a reputation for trustworthiness. Construal level, which refers to the level of abstractness or concreteness of our mental representations [38, 39], must then be a crucial factor in determining whether or not people recognize opportunities to invest in gaining a trustworthy reputation.

#### Construal levels and trust

According to Construal Level Theory [13, 40, 41], information can be represented or construed at various levels of abstraction or concreteness. High-level construals, which are relatively abstract, involve representations that are decontextualized and that allow for a long-term focus. In contrast, low-level, concrete construals are more contextualized and involve retaining more secondary details [13]. Cognitive abstraction (vs. concreteness) allows one to traverse mental distances [42]. According to CLT, engaging in cognitive abstraction can be likened to climbing to a higher spot (e.g., a tower). Construing information at more abstract (vs. more concrete) levels frees the mind to look further (i.e., “see the forest”), even though one may not be able to make out some of the finer-grained details (i.e., “see the trees”; [43]). For example, an abstract understanding of some prosocial behavior (e.g., helping) helps connect the behavior with its longer-term implications—gaining a trustworthy reputation—in a way that more concrete understandings of the same behavior (e.g., showing someone around, or giving feedback) do not [13, 39].

Hence, engaging in cognitive abstraction helps to expand one’s mental horizons or scope and effectively navigate towards more long-term goals [44]. In contrast, cognitive concreteness restricts one’s scope and thus makes people less effective in striving towards long-term goals [45, 46]. It should then be easier to see from the metaphorical high spot of a high-level construal than from the “ground floor” of a low-level construal the benefits of investing in establishing a trustworthy reputation by displaying prosocial behavior. More formally, building and maintaining a reputation for trustworthiness through the performance of prosocial behaviors requires the ability to traverse mental distances. One needs to be able to realize that performing prosocial behavior may later yield interpersonal benefits, and this requires one to traverse the distance between the now and the long-term. Additionally, one needs to be able to understand how the display of such behavior or the failure to do so may be perceived by people who are not in the room.

This argument leads to our first hypothesis:

*Hypothesis 1*: *Trustee’s cognitive abstraction (vs*. *concreteness) indirectly increases trust earned because cognitive abstraction (vs*. *concreteness) promotes trustee’s display of prosocial behavior*

Our argument implies that the effect of cognitive abstraction on the performance of prosocial behaviors should be particularly pronounced when such behavior is likely to yield reputational benefits for the person. The distinction between publicly visible behavior and privately performed behavior is relevant here. Specifically, displaying behavior publicly (vs. privately) is known to arouse concerns about the possible reputational consequences of the behavior [47–49]. This also applies to our current reasoning. In a setting in which third parties may be able to learn about one’s behavior (e.g., public settings), displaying prosocial behavior can boost one’s reputation as a trustworthy organization member; in a private setting, no such effects of displaying prosocial behavior can be expected. This reasoning leads to our second hypothesis:

*Hypothesis 2*: *The effect of trustee’s cognitive abstraction (vs*. *concreteness) on trust via their prosocial behavior*, *is observable when this behavior is performed publicly (rather than privately)*.

## Study 1

The consequences of received trust have mostly been studied in the context of the workplace. For this reason, our Study 1 was a survey conducted among employees of various organizations and their direct supervisors. Employees indicated their dispositional level of cognitive abstraction. Supervisors indicated their trust in the employee and the employee’s display of organizational citizenship behavior (OCB) was an indication of prosocial behavior [50]. We tested if the employee’s dispositional inclination to engage in cognitive abstraction (vs. concreteness) predicts increased trust from the supervisor to employee, and whether this relationship is mediated by the employee’s display of prosocial behavior (Hypothesis 1).

## Method

### Respondents and procedure

In the absence of established sizes of the effects of cognitive abstraction on prosocial behavior and trust in the literature, we estimated the effect to be medium-sized; we used the wp.mediation function from the *WebPower* package for *R* [51, 52] to determine a minimum sample size for detecting a medium-sized indirect (i.e., mediated) effect (i.e., *ab*_*cs*_ = .25, α = .05 and power = .80). The minimum sample size was 82. We recruited respondents from the independently managed, permanent research panel Flycatcher. Researchers have used this panel to collect data for research on organizational behavior [53, 54]. The quality of the Flycatcher panel is actively managed in manyways, one of which is removing from the panel respondents who provide low quality data more than once. We targeted panel members who were responsible for supervising at least one employee. Ethical approval for this study was obtained from the Review Board at the second author’s institution. All participants pre-consented to participate in scientific studies when they signed up at Flycatcher. Additionally, we asked for, and got, written explicit consent from all study participants.

We received 214 responses from supervisors. We asked the supervisors to provide the email address of one employee they supervised so we could ask the employee to respond to another questionnaire. Employees did not need to be panel members to be included. To stimulate employee participation, we raffled off among participating employees vouchers that could be used for online purchases. We checked the names and email addresses of the subordinates together with the supervisors’. Email addresses that we received from supervisors who provided suspicious information (e.g., an email address that might have been the supervisor’s own address instead of the employee’s) were not send an invitation to the study.

In total, 114 employees responded, so we included 114 matched supervisor–employee pairs in our analyses. See Table 1 for descriptive statistics for our sample. Matched supervisors did not differ significantly from unmatched supervisors in their mean scores on demographics, trust in the employee, and the reported level of employee’s OCB (all *F*s < 1, *p*s > .05, all η^2^ < .01). The correlations between these variables (all *p*s > .05) also did not differ significantly.

**Table 1:** Descriptive statistics for Study 1.

Variable	*M*	*SD*
Age employees	39.34	10.95
Age supervisors	43.95	12.02
Tenure employees (organization)	9.44	8.29
Tenure supervisors (organization)	11.53	9.46
Tenure employees (job)	7.07	6.30
Tenure supervisors (job)	8.00	6.72
Cognitive Abstraction	14.61	5.38
OCB	5.26	.79
Trust in Employee	5.82	1.00

*Note*: *N* = 114. Of the matched supervisors, 40 identified as female (35.1%), the rest as male. Of the employees, 48 identified as female (42.1%), the rest as male. In terms of educational attainment, 74 (64.91%) supervisors had a bachelor’s or master’s degree, 33 (28.94%) completed vocational education, and 7 (6.10%) indicated that they had a high school diploma but no other degrees. 59 (51.75%) Employees reported to have a bachelor’s or master’s degree, 35 (30.70%) indicated to have completed vocational education, and 20 (17.54%) indicated high school as their highest completed education.

In the above table, tenure refers to the number of years a respondent has worked at the same organization (tenure organization) or in the same job (tenure job), see below for the measures used to assess cognitive abstraction, OCB and trust.

#### Measures

Supervisors indicated their *trust* in the employee using a 4-item scale developed bySalamon and Robinson [55]. Example items are “I have faith in this employee’s integrity” and “I have faith in this employee’s benevolence” (1 = *not at all*, 7 = *very much so*).

Supervisors assessed the matched employee’s *OCB* using the 19-item OCB scale developed by [56]. Response options ranged from 1 (*strongly disagree*) to 7 (*strongly agree*). Example items are “This employee always goes out of his/her way to make newer employees feel welcome in the work group” and “This employee frequently communicates to co-workers’ suggestions on how the group can improve.”

We measured employees’ dispositional inclination towards *cognitive abstraction* (indexed by the employee) using [57] Behavioral Identification Form (BIF). The BIF iswidely used in construal level research [58–60]. The BIF contains 25 descriptions of actions at an intermediate abstraction level (e.g., “paying the rent”). Respondents who prefer the re-descriptions as higher-order goals (“maintaining a place to live”) rather than as lower-level means (“writing a check”) are disposed to construe events and objects in abstract (vs. concrete) ways. We summed up the number of selected higher-order goals options to form an index of cognitive abstraction (vs. concreteness). Higher scores represent a stronger inclination towards abstraction.

### Results

Table 2 presents the means, standard deviations, Cronbach’s alpha coefficients, and correlations among the study variables.

**Table 2:** Means, standard deviations, reliabilities, and correlations between Study 1 variables.

	Mean (*SD*)	1		2	2a	2b	2c	2d	3
(1) Cognitive Abstraction	14.605 (5.375)	.83							
(2) OCB	5.263 (.794)	.27 (.003)		.95					
(2a) Interpersonal Helping	5.375 (.929)	.23 (.014)		.90 (< .001)	.91				
(2b) Individual Initiative	5.139 (.875)	.24 (.011)		.86 (< .001)	.70 (< .001)	.88			
(2c) Personal Industry	5.362 (.936)	.25 (.008)		.87 (< .001)	.75 (< .001)	.63 (< .001)	.87		
(2d) Loyal Boosterism	5.178 (.918)	.24 (.012)		.85 (< .001)	.66 (< .001)	.65 (< .001)	.64 (< .001)	.89	
(3) Trust in Employee	5.823 (1.00)	.27 (.004)	.74 (< .001)		.74 (< .001)	.54 (< .001)	.74 (< .001)	.56 (< .001)	.96

*Note*. Cronbach’s α coefficients are presented on the main diagonal (KR-20 in the case of cognitive abstraction). Two-sided *p*-values are presented within brackets.

**Fig 2:**
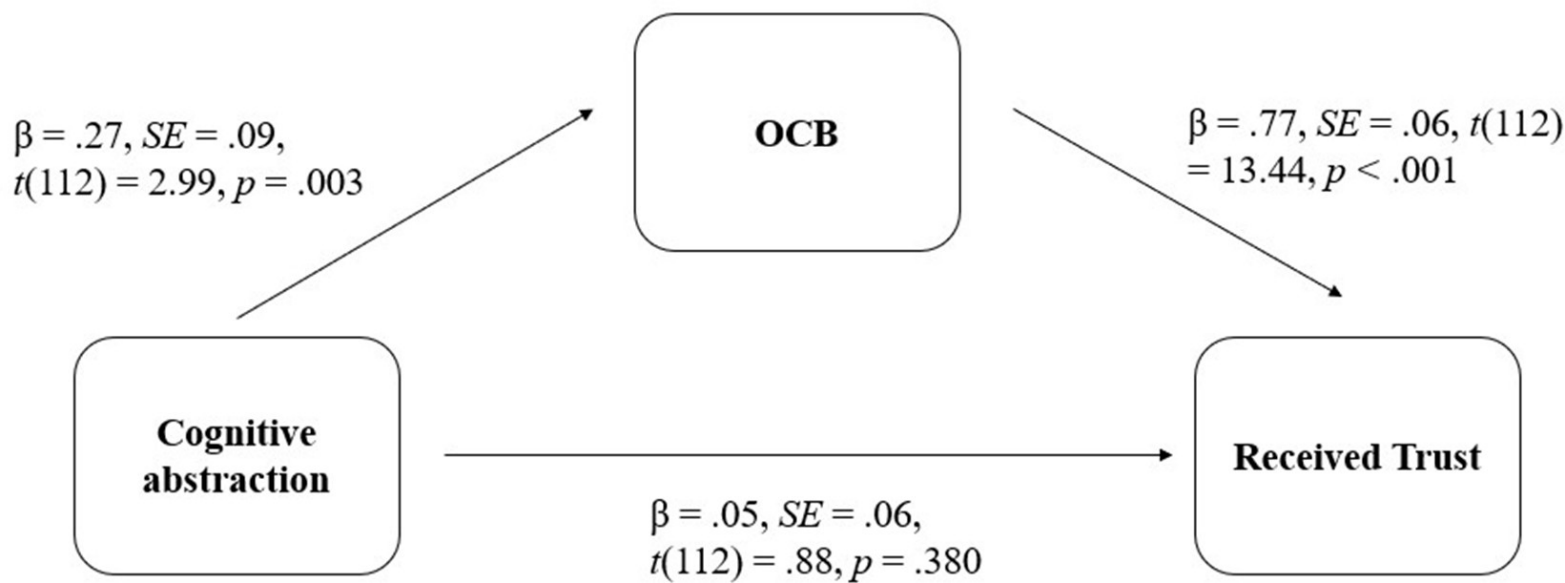
Path model for Study 1.

We used PROCESS macro for R to test Hypothesis 1 (model 4) [61], using 5000 bootstrap intervals as recommended by Hayes (2017); see Fig 2 below. Most importantly, this analysis revealed a significant indirect effect of cognitive abstraction on received trust via displayed OCBs, *point estimate* = .21, *BootSE* = .06, 95%CI[.09; .16]. The fact that cognitive abstraction and received trust were significantly univariately correlated in our data (*r* = .27, *t*(112) = 2.93, *p* = .004) but the direct effect of cognitive abstraction on trust was not (see Fig 2) is consistent with the mediational path that we proposed.

The OCB scale includes four subscales: interpersonal helping, individual initiative, personal industry, and loyal boosterism. Each subscale significantly mediated the relationship between cognitive abstraction and received trust. Additionally, controlling for employee’s and supervisor’s gender, age, organizational tenure, and education level do not change the direction or significance of any of the relationships reported.

Study 1 was a cross-sectional study. Hence, we could not draw causal conclusions from it [62], including conclusions about the mediational chain that we proposed [63]. We designed Study 2 to unambiguously test if induced cognitive abstraction (vs. concreteness) increases prosocial behavior and subsequently leads to increased trust from the interaction partner.

### Discussion of Study 1 and introduction to Study 2

In Study 1, we found that employees high (vs. low) in cognitive abstraction were trusted more by their supervisor, and this relationship was mediated by the employee’s performance of OCB. Study 1 thus supports Hypothesis 1.

Study 2 was a controlled experiment in which we used a yoked design. Using a yoked design provides for some advantages over more traditional designs when testing for mediation; for instance, reversed causality is not a potential problem nor is endogeneity due to common method variance. The yoked design means Study 2 consisted of two parts: Studies 2a and 2b. In Study 2a we induced variations in cognitive abstraction through a validated semantic induction procedure [64] and provided participants with an opportunity to help another participant. Each participant in Study 2b indicated their trust in their partner, who was a matched participant from Study 2a, based on information about the latter’s prosocial behavior. In both studies, participants believed they interacted with their matched partner online [37, 65] on the Prolific platform.

## Study 2

### Method

#### Participants

In this study, we estimated a mediation model in which both the mediator and the dependent variable are count variables. We used the ssMediation.VSMc.poisson function from the R-package powerMediation [66, 67] to estimate the required sample size. We used the effect size found in Study 1 (that is, *ab*_*cs*_ = .22) as estimate of the true effect size. These analyses indicated that we needed 123 participants to detect an indirect effect of *ab*_*cs*_ = .22, with power = .80 and α = .05.

We recruited participants for Studies 2a and 2b from Prolific (https://prolific.ac/). Participants received £1.00 ($1.31 at the time) for their participation. In total, 135 Prolific members participated in Study 2a. One of them did not consent to the conditions, four others abandoned the study halfway. We were then left with an *N* of 130 (*M*_*age*_ = 32.30 years, *SD* = 12.14), 66 (50.07%) of which identified as female, 63 (48.46%) as male, and 1 (.76%) as another gender, see Table 3, below, for a balancing table of descriptive variables per condition. We recruited a non-overlapping sample of 130 participants for Study 2b (*M*_age_ = 36.46 years, *SD* = 13.43), 54 of which identified as female (41.54%), 74 (57.92%) as male, and 2 (1.54%) as another gender. We administered an attention check for both samples we recruited, in the 2a sample, 119 participants provided the correct answer, in the 2b sample, 114. We did not filter out any participants, based on the attention check, however. No abnormal response patterns were observed in either sample.

**Table 3:** Descriptive statistics by experimental condition in Study 2a.

	Low CL condition	High CL condition	Significance
Age	*M =* 36.36, *SD* = 14.08	*M =* 36.36, *SD* = 12.87	*F* < .001, *p* > .99
Gender	28 men, 35 women, 1 other	26 men, 34 women, 1 other	*t* = .13, *p* = .90

*Note*: p-value for age based on one-way ANOVA, for Gender on ordinal logistic regression

Ethics approval for the procedures of this study, both part a and b, was obtained from the Review Board at the second author’s institution. Written consent was obtained from all Study 2a and b participants before they entered their respective parts of the study.

#### Procedure of Study 2a: The effect of cognitive abstraction on prosocial behavior

Study 2a participants learned that they were to complete a number of new tests that we had ostensibly designed for use in assessment centers. We also informed them that other participants were going to later assess their performance on these tests. We used this cover story to establish a relationship between participants in Studies 2a and 2b that would be convincing to participants in Study 2a [37].

We then conveyed to Study 2a participants that they could first complete a “warm-up exercise” before they start the tests. In fact, this exercise was our cognitive abstraction induction procedure. Specifically, we used the commonly used why-vs.-how priming procedure developed by [64]. This procedure reliably induces variations in cognitive abstraction [68, 69]. Participants were prompted to consider behaviors described at an intermediate level of abstraction (e.g., “maintain and improve your health”). They were then prompted to consider either *why* they would engage in such behavior (abstract condition) or *how* they would engage in that behavior (concrete condition). This was repeated four times for the prompt “maintaining and improving your health” and four times for the prompt “dressing well” [70].

To check the cognitive abstraction manipulation, we coded participants’ responses to the prime with the Linguistic Category Model (LCM) dictionary [71]. The LCM dictionary assigns an abstraction score on the basis of the type of words used: adjectives like “healthy” are considered more abstract, while action verbs like “eating” are considered more concrete [72]. The LCM is considered a state-of-the-art approach for measuring cognitive abstraction [73–75]. One-way ANOVA indicated that participants in the cognitive abstraction condition used more abstract words (*M* = 34.51, *SD* = 5.63) in their responses than participants in the cognitive concreteness condition (*M* = 30.39, *SD* = 5.63, *F*(1, 132) = 18.00, *p* < .001, η^2^ = .12).

Subsequently, Study 2a participants were informed of “a software problem” that prevented us from presenting the assessment center tests to them. Before sending them to the end of the study, we told the participants the following (our cover story): the other participant, who was going to check their performance, could use some help solving some anagram puzzles; any puzzle they solved would no longer be solved by their partner; helping was completely voluntary; and agreeing or not to help their partner would neither increase nor reduce their remuneration. We made sure that the participants understood that solving more anagrams would benefit their partner, not the researchers, and that let their partner know the number of anagrams they attempted to solve. Several studies have shown that emphasizing the public nature of behavior is sufficient to make reputational concerns salient [31, 76, 77]. We used the number of anagrams that participants attempted to solve (*M* = 6.07, *SD* = 10.31) as an index of participants’ prosocial behavior. After completing the task, we thanked the participants and fully debriefed them. Nobody objected to the procedures followed.

#### Procedure of Study 2b: The effect of prosocial behavior on trust

We assigned each participant in Study 2b randomly to receive information about the number of anagrams one Study 2a participant had tried to solve and the average number of anagrams all Study 2a participants had tried to solve (as a benchmark). We did not inform participants in Study 2b about any other aspect of the procedure of Study 2a.

Using a trust game [78], we measured the effect of prosocial behavior on the extent to which Study 2b participants trusted the yoked participant from Study 2a (their partner),. At the end of the study, we organized a raffle in which Study 2b participants received ten lottery tickets that gave them ten chances to win gift vouchers worth €50.00-. They were offered the opportunity to share their tickets with their partner. We told them that the number of tickets they shared would be tripled before these were transferred to their Study 2a partner and that the latter would be offered the opportunity to give back to them some or all of the received tickets. In this way, sharing tickets would make the outcomes of Study 2b participant vulnerable: their outcomes would increase or decrease depending on their partner’s decision (Study 2a participant) to whether send back more or lesser tickets than they had originally transferred [32]. Since we were only interested in the trust that Study 2b participants had in their partner, we no longer involved Study 2a participants at this stage; instead, we gave all the participants in Study 2b an equal chance to win the raffle. The number of tickets that participants in Study 2b decided to share with their partner constituted our measure of trust (min = 0, max = 10, *M* = 5.15, *SD* = 2.53; [79]).

Before we asked the participants how many tickets they would share, we made sure they understood the procedure of the trust game and the implications of their decision. We asked three comprehension questions (i.e., “How many tickets did you initially receive?,” “Say that you share all your tickets with your partner, how many tickets does he/she then receive?”, and “Say, you invested all 10 tickets, and your partner decides to return half of the amount s/he received. How many tickets do you receive from your partner in the end?”). When participants provided an incorrect answer to a question, we corrected them and again explained the structure of the game.

Finally, we collected demographic information and fully debriefed participants.

### Results

#### Study 2a hypothesis test

Using negative binomial regression by means of the glm.nb function from the *R*-package MASS [80], we tested the effect of cognitive abstraction on the number of anagrams that participants attempted to solve. Participants in the abstract condition attempted to solve significantly more anagrams (*M* = 7.59, *SD* = 12.43) than participants in the concrete condition did (*M* = 4.07, *SD* = 6.99, *b* = .31, *SE* = .14, *t* = 2.22, *p* = .028, *IRR* = 1.37).

#### Study 2b hypothesis test

To check whether prosocial behavior affected helpfulness perceptions, we asked participants in Study 2b to indicate on a scale of 0 (*not helpful at all*) to 10 (*very helpful*) how helpful they thought their partner was (*M* = 4.34; *SD* = 3.83). Negative binomial regression revealed that participants in Study 2a who solved more anagrams were perceived by their partner in Study 2b as more helpful (β = .20, *SE* = .02, *t*(129) = 8.61, *p* < .001).

Negative binomial regression revealed a significant positive effect of the number of anagrams Study 2a participants attempted to solve on the number of tickets shared (*b* = .02, *SE* = .01, *z* = 2.32, *p* = .020, *IRR* = 1.02)

#### Indirect effect analyses

**Fig 3:**
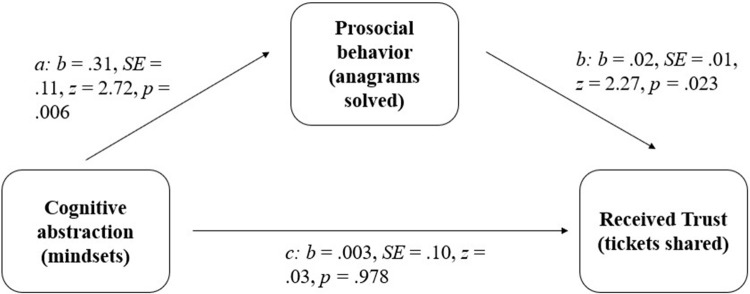
Path model for Study 2 (parameter estimates based on negative binomial regression).

The above presented analyses revealed the following: (1) participants displayed more prosocial behavior in the abstract mindset induction condition than in the concrete mindset induction condition (Study 2a), and (2) more helpful participants were trusted more (Study 2b). We used indirect effect analyses to test our prediction that participants in the abstract (vs. concrete) mindset induction condition in Study 2a would be trusted more by their linked participant in Study 2b because participants in Study 2a showed higher levels of helpfulness (i.e., tried to solve more puzzles). We used the *R*-package *mediation* [81] to do these analyses. We found support for Hypothesis 1 (see Fig 3, below): there is a significant indirect effect of cognitive abstraction (vs. concreteness) on trust via trustee’s helpfulness (*point estimate* = .15, 95% CI [.07; 0.25], *p* < .001; we found an indirect effect of similar size when filtering out participants who failed our attention check in Study 2a, point estimate = .14, p < .001. Since the mediation package does not support negative binomial regression, we based our estimates of the a- and b-paths (i.e., the path from IV to mediator and from mediator to DV, respectively) on Poisson regression coefficients.

#### Discussion of Study 2 and introduction to Study 3

The results of Study 2 corroborate those of Study 1: participants in an abstract (vs. concrete) mindset were more likely to help their partner and were therefore trusted more by their partner. Together, Studies 1 and 2 provide evidence high in ecological and internal validity for Hypothesis 1.

The purpose of Study 3 was to evaluate whether the effect of the trustee’s cognitive abstraction (vs. concreteness) on the trust they received, via trustee’s prosocial behavior, is observed when this behavior can be performed publicly (rather than privately). In Studies 1 and 2, prosocial behaviors took place in a public setting. In Study 3 we manipulated whether prosociality would be publicly visible (as in Studies 1 and 2) or if it would be private. As in Study 2, we used Prolific because members of this platform are very much aware of the importance of their reputation with researchers.

We induced variations in cognitive abstraction in Study 3a by manipulating the temporal distance between the here and now of participants and an opportunity to act upon their expressed prosocial intent towards one’s partner (i.e., either in the near or distant future). Participants in Study 3b indicated their trust in their partner (a matched participant from Study 3a) based on information about the latter’s expressed prosocial intent. Varying temporal distance is often used to induce variations in cognitive abstraction vs. concreteness [41, 82]. Participants need to engage in cognitive abstraction to mentally represent a distant (vs. close) target (i.e., an opportunity to help, in this case) because representing more distant targets requires more decontextualized (i.e., high-level or abstract) construal [43, 83]. In Study 3, we focused on expressed prosocial intent within a temporal distance setting for two reasons. First, expressing prosocial intent is highly relevant for building and maintaining trust [84]. Second, there is commonly temporal distance between the moment when one commits to a certain act and the opportunity to act upon that commitment; for instance, when agreements are made about an employee’s future performance following a performance appraisal.

## Study 3

### Method

#### Participants

Like in Study 2, we estimated in Study 3 a mediation model in which both mediator and dependent variable displayed are count variables; however, this time, we set out to detect an interaction effect on the first path (from independent to mediator) in addition to an indirect effect (see Fig 1). Similarly, we used the ssMediation.VSMc.poisson function from the *R*-package powerMediation [66] to determine that we needed at least 164 participants to detect an effect of *ab*_*cs*_ = .22 with α = .05 and 1 – β = .80.

For Study 3a, we recruited 300 participants through Prolific (*M*_age_ = 31.00, *SD* = 11.28). Of these, 146 identified as female (48.7%), two as another gender (.7%), one declined to answer this question (.3%), and the rest (50.3%) identified as male. For Study 3b, we recruited a nonoverlapping sample (*M*_age_ = 32.38, *SD* = 11.14), of which 149 identified as female (49.7%), 2 as another gender (.7%), and the rest as male (49.7%). We excluded from participating in Study 3 Prolific participants who already participated in Study 2. See Table 4, below, for a balancing table per experimental condition.

**Table 4:** Descriptive statistics by experimental condition in Study 3a.

	*Public conditions*		*Private conditions*		
	Low temporal distance	High temporal distance	Low temporal distance	High temporal distance	Significance
Age	*M =* 32.88, *SD* = 11.49	*M =* 33.89, *SD* = 12.08	*M =* 32.20, *SD* = 11.13	*M =* 32.95, *SD* = 10.54	*F =* .45, *p =* .50
Gender	30 men, 44 women, 0 other	37 men, 39 women, 0 other	42 men, 36 women, 1 other	37 men, 32 women, 1 other	*t* = .73, *p* = .46

*Note*: p-value for age based on one-way ANOVA, for Gender on ordinal logistic regression

Ethics approval for the procedures of this study, both parts a and b, was obtained from the Review Board at the second author’s institution. Written consent was obtained from all Study 3a and b participants before they entered their respective parts of the study.

#### Design, procedure, and measures Study 3a: The effect of temporal distance and confidentiality on expressed prosocial intent

We assigned participants randomly to one of four conditions that resulted from orthogonally manipulating temporal distance (near vs. distant) and confidentiality (public vs. private helping).

We followed the same procedures as in Study 2a, with two differences. First, instead of inducing variations in cognitive abstraction by means of a priming procedure, we varied temporal distance. In the temporally *distant* condition, participants learned that Study 2b would run in about a year; in the temporally *near* condition, participants learned that Study 2b would run in about a week. We modelled this manipulation on existing temporal distance manipulations found in the cognitive abstraction literature [85, 86].

We tested whether temporal distance induced variations in cognitive abstraction. Immediately after participants had responded to the temporal distance question (i.e., the manipulation check question) but before they had to decide whether to help their partner or receive information relevant to the confidentiality manipulation, we asked participants to write a short text about their first impressions of their partner participant. We gave participants some prompts (e.g., “What gender do you think they are?,” “How old do you think he or she is?”), and we coded these responses using the LCM dictionary, as in Study 2. Before we automatically coded the texts, a research assistant blind to our hypotheses checked the responses to see whether they contained adjectives and/or verbs, which the LCM coding scheme codes for (Semin & Fiedler, 1988). Seven participants did not write any text, while 33 (11%) did but they did not use adjectives or verbs (e.g., “Female, 18, computer, Holland, European”). We excluded these participants, reducing our effective *N* for this analysis to 260. (In Study 2, all responses were of sufficient length to be coded because we used LCM to evaluate responses to the cognitive abstraction prime, for which participants had to write eight different responses.) One-way ANOVA revealed that participants in the temporally distant condition used more abstract words (*M* = 19.02, *SD* = 6.85) than participants in the temporally close condition (*M* = 16.96, *SD* = 7.31, *F*(1, 259) = 5.51, *p* = .020, η^2^ = .02).

The second difference was we manipulated confidentiality by explicitly informing participants whether details about their behavior would be shared with their partner from Study 3b and the study leaders. In the *public* condition, we gave participants the same information as in Study 2a: i.e., information about their behavior would be logged and would be communicated to all potential partners in Study 3b. In the *private* condition, we told participants, “Your actions during this study will not be observed in real time by one of your fellow participants. This means that all your actions will be completely anonymous.” We also told them that while anonymized data would be made available to the researchers, identifying information would be retained in the system such that behavioral choices could not be traced back to them even by the research team. Several studies have shown that publicly (vs. privately) displaying behavior makes reputational concerns salient [31, 77, 87, 88]. In fact, publicly (vs. privately) displayed behavior increases prosociality even when only passive observers (i.e., ones who can only observe but not reward, punish, or evaluate the actor) are present [16]. One reason can be traced to our evolutionary history: In the ancestral environment, one’s reputation could mean the difference between death and survival. As such, a generalized concern with reputation has become internalized in our motivational bases [89]. Furthermore, reputation has been found to be very salient concern for participants recruited from online platforms [90].

As described in the next section, we used expressed prosocial intent as outcome variable in Study 3a (and therefore, the mediator in Study 3 overall). More specifically, we asked participants to what extent they would be willing to help their partner (participant from Study 3b) during the time they believe the latter study would be running (i.e., either relatively soon or relatively far away in the future). We informed participants that the estimated study length of Study 3b would be an hour and asked them to indicate the number of minutes they would be willing to help, *M* = 9.67, *SD* = 15.12.

#### Procedure and measures Study 3b: The effect of expressed prosocial intent on trust

We coupled each participant in Study 3b with one other participant from Study 3a (hereon, partner). We informed the former about the number of minutes their partner had indicated to be willing to help, the average number of minutes that participants in Study 3a were willing to help, and whether their partner was aware that this information would be shared with them. As in Study 2, participants were asked how many of the 10 tickets they would be willing to share as trustor in a trust game (*M* = 5.10, *SD* = 3.48).

### Results

#### Study 3a hypothesis test

**Fig 4:**
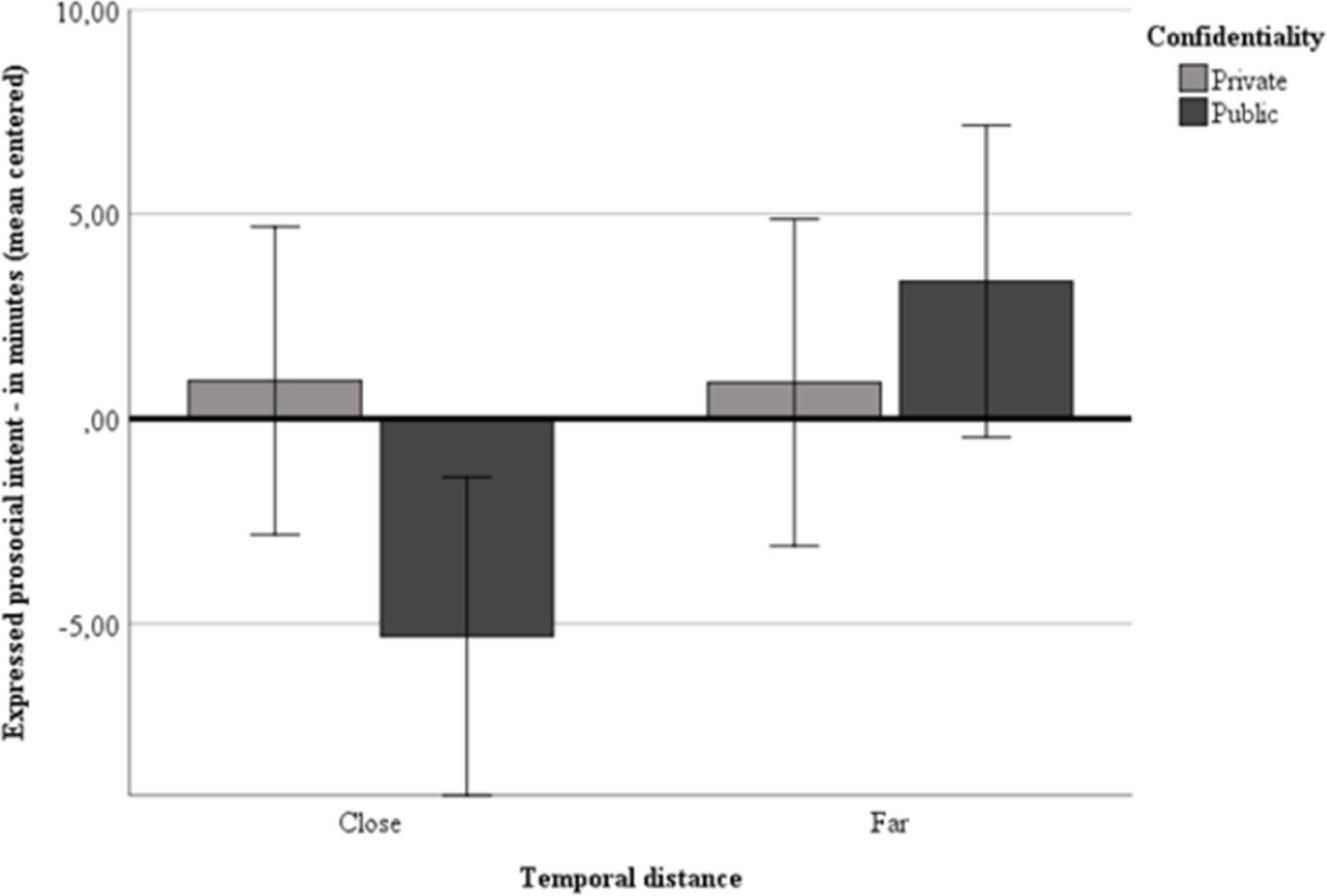
Interaction effect of temporal distance and confidentiality on expressed prosocial intent in Study 3. Error bars represent 95% CIs around the mean.

Like in Study 2, we used the glm.nb function from the *R*-package MASS [80] to fit a negative binomial regression model to these data. This model revealed significant main effects of both temporal distance (*b* = .15, *SE* = .02, *z* = 10.13, *p* < .001, *IRR* = 1.17) and confidentiality (*b* = -.08, *SE* = .02, *z* = -5.57, *p* < .001, *IRR* = .92) on prosocial intent. More importantly, we found a significant Temporal Distance × Confidentiality interaction effect (*b* = .15, *SE* = .02, *z* = 10.22, *p* < .001, *IRR* = 1.17; see Fig 4). In support of Hypothesis 2, simple effects analyses revealed that high (vs. low) temporal distance significantly increased prosocial intent in the public condition (*b* = .61, *SE* = .04, *z* = 13.71, *p* < .002) but not in the private condition (*b* = -.002, *SE* = .04, *z* = .07, *p* = .948)–see Fig 4 below for a graphical depiction of this interaction.

#### Study 3b hypothesis test

As a check, we asked participants in Study 3b to indicate on a sliding scale (min = 0; max = 10) how helpful they found their partner to be (*M* = 5.84, *SD* = 3.75). Participants in Study 3a who indicated to be more willing to help were rated as more helpful by their partner in Study 3b (β = .14, *SE* = .01, *t*[299] = 15.59, *p* < .001).

Negative binomial regression revealed a significant effect of prosocial behavior on tickets shared (*b* = .02, *SE* = .002, *z* = 6.69, *p* < .001, *IRR* = 1.02). As suggested by one of our anonymous reviewers, we additionally assessed whether the difference in the perception about the prosocial intent of study 3a participants by confidentiality is conditional on the number of minutes study 3a participants are willing to help. OLS regression revealed a significant main effect of the number of minutes study 3a participants are willing to help, β = .68, SE = .04, t(295) = 15.48, p < .001, but no significant main effect of conditionality, β = -.02, SE = .04, t(295) = -.35, p = .724 or a significant interaction between these two variable, β = .03, SE = .04, t(295) = .78, p = .438. We thus find no evidence that conditionality affected perceptions of helpfulness.

#### Indirect effect analyses

**Fig 5:**
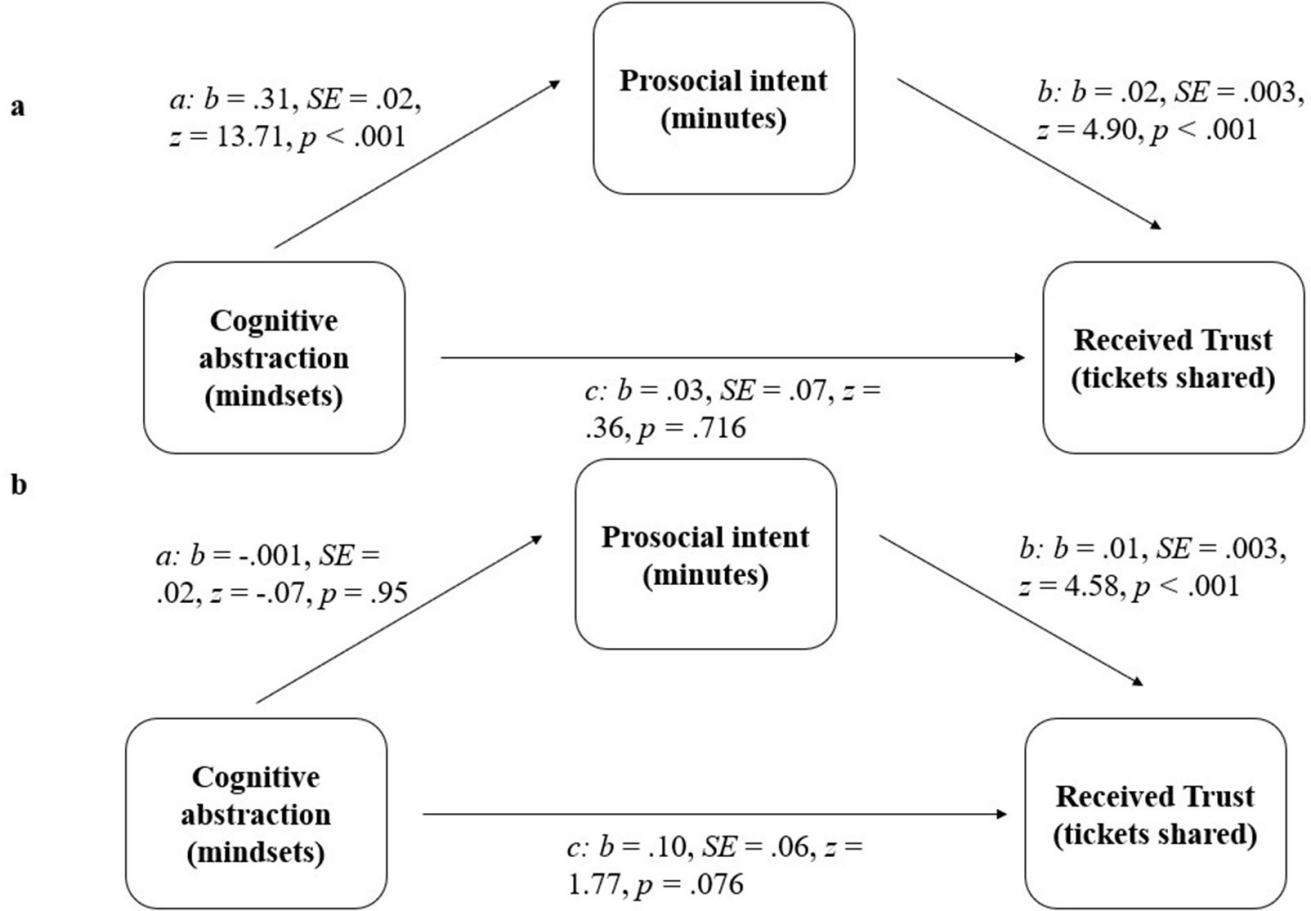
Path models in the public (a, above) and the private (b, below) conditions in Study 3.

We used the R-package mediation [81] to test for mediation. We found a significant indirect effect of temporal distance, via expressed prosocial intent, on trust in the public condition (*point estimate* = .42, 95% CI[.29;.57], *p* < .001). However, we did not find a significant indirect effect in the private condition (*point estimate* = -.001, 95% CI[-.07;.07], *p* = .996). We found broadly similarly sized indirect effects when filtering out those participants who failed our attention check questions, public condition: *point estimate* = .35, *p* < .001; private condition, *point estimate* = .003, *p* = .420. Moreover, the indirect effect in the public condition was significantly different from the effect in the private condition (*point estimate* = .38, 95% CI[.27;.51], *p* < .001), thus indicating moderated mediation (see Fig 5A and 5B).

## General discussion

We showed across three studies that cognitive abstraction (vs. concreteness) facilitates investment in gaining a trustworthy reputation through the display of prosociality. We found this effect in an organizational field study that is high in ecological validity (Study 1) and in two experiments that allow for causal conclusions about the indirect effect (Studies 2 and 3). We found the effect when we operationalized cognitive abstraction (vs. concreteness) as trait-level individual differences in abstraction (Study 1), as induced variations in abstraction (vs. concreteness) (Study 2), and as induced variations in temporal distance from the opportunity to perform prosocial behavior (Study 3). We found support for the mediating role of trustee prosocial behavior when we operationalized it as OCB (Study 1), as helping behavior (Study 2), and as expressed prosocial intent (Study 3).

Study 3 also showed *when* cognitive abstraction (vs. concreteness) makes one act more prosocially. The effect of cognitive abstraction on this type of behavior and the subsequent trust one receives is limited to situations where that behavior has reputational consequences (i.e., when the display of prosociality is public rather than private).

### Theoretical contributions

One of the contributions of our research is to the literature on trust. Given that being trusted has many positive implications for one’s wellbeing [1–3, 6, 30, 91], scholars have suggested that people actively attempt to build or preserve trust [8–10]. However, it is not clear when and why people invest in actions that may gain them trust. More specifically, extant literature has mostly focused on factors that motivate people to invest in being trusted, such as anticipated guilt [8]. Our results complement this stream of research by showing that in addition to being motivated to engage actively in building trust, people also need to be cognitively able to spot opportunities for doing so (i.e., when they construe the situation on higher vs. lower, levels).

Our research also contributes to the literature on the role of cognitive abstraction in interpersonal, group, and organizational settings. As recognized in a recent review of this literature, a major impediment to understanding effects of abstraction (vs. concreteness) in such contexts is that it is unclear how abstraction relates to interpersonal trust [20]. Unfortunately, and rather confusingly, scholars have speculated that abstract *and* concrete cognition may heighten the trust one receives [92, 93]; but little empirical evidence has been provided for either direction. We offer a clear rationale (and empirical support) that is based on construal level theory for why trustees in an abstract (vs. concrete) mindset are more likely to gain trust: because cognitive abstraction (vs. concreteness) facilitates the enactment of behavior that gains one trust (i.e., prosocial behavior) in circumstances that are most conducive to trust building (i.e., contexts in which behavior is likely to have reputational consequences).

Cognitive abstraction involves traversing mental distances [46, 94], such as between self and others. Therefore, it has been argued that abstraction should increase prosociality [22]. However, evidence for this claim is rather indirect, showing only that cognitive abstraction (vs. concreteness) makes people apply justice principles to distant others [22], increases the anticipated reward of enacting prosocial behavior [16], and strengthens prosocial attitudes [23]. In fact, some prior findings suggest connections between concreteness, rather than abstraction, and antecedents of prosociality (e.g., empathic concern; [95]). Hence, our findings provide much needed support for the conjecture that cognitive abstraction facilitates prosocial *behavior* [21].

Furthermore, our research goes beyond just showing *that* cognitive abstraction makes one more likely to engage in prosocial behavior; it also shows *when* and *why* this is the case. Study 3 showed that cognitive abstraction (vs. concreteness) does not facilitate prosocial behavior just for the sake of it (i.e., it does not increase prosocial motivation per se). If that were the case, then cognitive abstraction (vs. concreteness) would have increased prosociality in private as well. Instead, abstraction only increases *public* displays of prosociality, relative to concreteness. Scholars interested in the interpersonal or organizational consequences of cognitive abstraction would do well to consider whether reputation concerns are relevant in the situations under study.

### Practical implications

One of the practical implications of our results is that employees and managers or athletes in team sports interested in furthering their reputation in their organization/teams should realize that engaging in cognitive concreteness, rather than abstraction, may cause them to miss opportunities to promote their reputation among peers. Engaging in concreteness, rather than thinking about the long-term consequences of one’s behavior, has been known to make people more focused on completing the task at hand [54]. This is especially problematic for those who are disposed towards concrete, rather than abstract, cognition or who have roles that require concrete cognition (e.g., jobs that require one to be focused on details). Such individuals may be better off switching towards a more abstract way of thinking whenever they interact with team or organization members so they can more easily recognize opportunities for gaining trust [96].

On a collective level, hiring employees or selecting athletes who tend to engage in abstract (vs. concrete) cognition may increase the prevalence of prosocial behavior within the organization or team and may lead to higher levels of trust within the organization or team. People who are disposed to abstract (vs. concrete) cognition are more likely to display prosocial behavior and are trusted more (Study 1). However, focusing only on cognitive abstraction in recruitment and selection is insufficient to stimulate prosocial behavior and trust if tasks are organized such that they induce concrete (rather than abstract) cognition. One factor that organizations and teams should therefore carefully manage is time (which we manipulated in Study 3). For example, setting many tight deadlines likely induces a focus on the here and now—and thus concrete cognition—even for the most chronically abstract minded [97]. Setting tight deadlines may thus reduce the incidence of other-regarding behavior within the organization or team, and therefore, of interpersonal trust.

At the same time, it should be noted that abstract (vs. concrete) cognition is relevant to prosocial behavior and trust building only in situations where people’s reputation is at stake. Abstract (vs. concrete) cognition thus does not make people intrinsically more prosocially motivated nor intrinsically more trustworthy. In fact, abstraction may also make people more hypocritical in their prosocial displays, as in situations in which “cheap talk” is sufficient to reap reputational benefits [92]. Hence, work organizations or sports teams that require members to follow through on their prosocial commitments and not let people get away with not delivering on their promises will see more benefits from the kind of interventions we propose in the preceding paragraph.

### Limitations and suggestions for further research

Our research has some limitations. Although the results from Study 1 are consistent with the proposed mediational chain, the cross-sectional nature of this study means we cannot draw conclusions about the causal ordering of the variables. Studies 2 and 3 provide a much stronger way to assess causality. However, here, our conclusions about mediation hinge on two assumptions [63]. First, we assumed that the effects of cognitive abstraction on prosocial behavior and of prosocial behavior on received trust are linear. Second, we assumed that cognitive abstraction does not interact with prosocial behavior to predict received trust.

Another limitation pertains to manipulating cognitive abstraction (vs. concreteness) by inducing variations on temporal distance in Study 3. People prefer immediate over later rewards, and later over immediate effort [98, 99]. Participants might have preferred to help others (an effort) later rather than sooner, which would suggest that the results of Study 3 could not be (fully) explained by variations in cognitive abstraction but by time preferences. However, Study 1 and 2 operationalized cognitive abstraction (vs. concreteness) directly, without such ambiguities being present. Furthermore, Study 3 showed that reputational concerns shape people’s behavior: In the public condition, participants expressed to be more willing to help in the distant than in the near future. In the private condition, we did not find any effect of temporal distance.

Future research may build on the present findings. We argued that because cognitive abstraction (vs. concreteness) helps people see how their behavior is connected to their long-term goals, it makes them display prosocial behavior to gain a trustworthy reputation. The benefits from such a reputation tend to accumulate over time. This suggest that other variables may also facilitate the display of prosocial behavior to gain trust, most clearly, long-term orientation. This variable refers to “the cultural value of viewing time holistically, valuing both the past and the future rather than deeming actions important only for their effects in the here and now or the short term” [100]. Based on this definition, measures of long-term orientation include “tradition” (looking at the past) and “planning” (looking at the future; [100]). Abstract (rather than concrete) cognition may facilitate the planning element of long-term orientation. Future research might find that people who score highly on planning display more prosocial behavior to gain more trust.

Our results show that the positive effect of abstraction (vs. concreteness) on prosocial behavior is limited to situations with reputational relevance. These results leave open the possibility that, for instance among individuals with a strong proself orientation [101], displayed prosociality may eventually result in their exploiting their interaction partner’s vulnerability once they have gained the trust. Among some self-oriented individuals, exploitation could be a long-term goal and having a trustworthy reputation could be a means to achieve this goal [102]. Thus, subject to specific trait-level moderators, abstraction may make some people less trustworthy in the long run. We expect this to be the exception rather than the rule given trust’s great value as a social resource [29]. Still, the question whether cognitive abstraction (vs. concreteness) facilitates long-term trustworthiness should be a subject of further research.

Furthermore, people may be concerned with different forms of reputation. For example, one may be particularly interested in establishing a trustworthy reputation with a specific other (e.g., one’s teacher at school, or one’s supervisor at work). Others, however, may be more focused on establishing their reputation within a broader community (e.g., one’s school or work organization; [7]. From a construal level perspective, the latter form of reputation is a more abstract goal than the former because the school or organizational community is a more abstract target than a specific person. Hence, an interesting research question is whether the benefits of cognitive abstraction in terms of a trustworthy reputation are as pronounced when one specifically focuses on a specific other vs. on the wider community.

### Concluding remarks

Our ability to mentally traverse time and space through cognitive abstraction aids the building and maintenance of trusting relations through the performance of prosocial behaviors within interpersonal, group, and organizational settings. It does so, however, *not* because cognitive abstraction (vs. concreteness) makes people necessarily more prosocially motivated, but because it makes people more sensitive to reputational concerns.
